# Editorial

**Published:** 1903-10-15

**Authors:** 


### EDITORIAL.

ALMOST every new State law that has been enacted within the past five years has contained a clause which requires that all persons desiring to practice dentistry shall be compelled to pass an examination before the State Examining Board can issue a license. This comes about because the colleges have thrown on the market each year an increasing number of competing dentists, and these graduates must necessarily find business even at the expense of practitioners long located in favored circumstances. It may also be possible that the State Examining Board members have become convinced that they are capable of determining by a two or three days' contact with a student whether or not he is fit to practice intelligently the profession of dentistry. A college faculty often finds it difficult to decide this question after three years of constant oversight of the behavior of these men.

There are evidences now appearing of a reaction from a new source, however. It is being found that it is high time some change is made which will do away with examinations for such as may have been licensed in one State and desire to remove to another. Interstate reciprocity is the remedy now advocated to cure this evil. Will it suffice? Possibly it will help, but the probabilities are that it will fail to accomplish the object, for the reason that it will require universal concurrence of the various States, and this is a condition hardly to be expected. It is too long and tedious a process to be really practicable. We predict that at no distant day this effort will be abandoned, and we shall return to the old and satisfactory standard of recognizing the graduate of a reputable collegiate institution as competent to register without examination for practice in any State.

We would not say that dental colleges have not abused the public confidence in times past by sending out as graduates incapable men; but we conscientiously believe that the dental college as conducted to-day is more likely to suitably prepare dentists than are preceptors and State Examining Boards. A case recently came under our observation of a young man who had never spent a day in a dental college or dental office in acquiring technical skill who went before a State Examining Board and passed its examination with a high standing, whilst several college graduates failed on the same examination. This would seem to reflect adversely on the college as a means of preparing students for practice, and perhaps it does; but the probabilities are that the examining board had no proper conception of its duty or business as viewed from an educational standpoint. By maintaining such a standard the public will be deprived of that intelligent skill which it is its right to have at command. Education is not a process of cramming facts, but a process of mental, and, in our case, of technical discipline. A man with a remarkable memory may appropriate an unabridged dictionary or a quiz compend and still be unable to correlate it for any useful purpose.

It is no idle boast to say that dental graduates are better trained to-day than ever before, and with the new requirements of a four years' course there will come still greater improvements in methods and results, notwithstanding the statement a prominent State Board Examiner made recently that the dental colleges had taken a foolish position in increasing the length of the course. We believe that time will demonstrate that the college standard is safer than any State Board.

